# Supplementary material for: Circulation of hepatitis delta virus and occult hepatitis B virus infection amongst HIV/HBV co-infected patients in Korle-Bu, Ghana
Source: PLoS One. 2021 Jan 7;16(1):e0244507. doi: 10.1371/journal.pone.0244507 (PMC7790253; doi:10.1371/journal.pone.0244507)
Supplement: S1 Questionnaire — (PDF) [file pone.0244507.s001.pdf]

HDV SURVEY QUESTIONAIRE FORM

Sample ID: ………………… Age: ……yrs

Sex: M ☐ F ☐ Hospital folder Number: ………………

Nationality: …………………… Date of Diagnosis: ……………

Date 1st blood sample taken: …………… Date 2nd blood sample taken: ……………

Diagnosis: Acute Hepatitis B ☐ Chronic Hepatitis B ☐

Hepatocellular Carcinoma ☐ Liver Cirhosis ☐

LFT results: ALT……….. AST………..

Total Bilirubin…………. Direct Bilirubin………..

Type of ART: ……………………………………………………………………………

Duration of ART: ………………….

Contact of Interviewer

Mobile No. ………………………….. E-mail:…………………………………………..

NMIMR INSTITUTIONAL REVIEW BOARD
VALID UNTIL
10 MAY 2019
APPROVED DOCUMENT
