# Supplementary material for: Circulation of hepatitis delta virus and occult hepatitis B virus infection amongst HIV/HBV co-infected patients in Korle-Bu, Ghana
Source: PLoS One. 2021 Jan 7;16(1):e0244507. doi: 10.1371/journal.pone.0244507 (PMC7790253; doi:10.1371/journal.pone.0244507)
Supplement: Attachment — Submitted filename: Response to reviewers_9122020.doc [file pone.0244507.s003.doc]

**PONE-D-20-18263**

**"Circulation of hepatitis delta virus and occult hepatitis B virus infection amongst HIV/HBV co-infected patients in Korle-Bu, Ghana"**

**Letter responding to comments:**

We are pleased that PLOS ONE will consider a review of our manuscript pending satisfactory revisions as suggested by the editor-in-chief and the reviewers. We are grateful to you for your constructive comments. We explain in detail below how we have addressed the concerns and have indicated the page and lines of the revised text in the accompanying revised manuscript.

**Reviewer #2 Comment**

| **Comment** | **Author Response** | **Lines** |
| --- | --- | --- |
| Reviewer #2: I find some inconsistencies in the results presented in Table 3 on Demographic Characteristics of Study Participants regarding ART. Page 9 line 176 states that two patients were ART naive (out of the 113 study participants). However, in Table 3, under duration of ART, a total of 112 patients are indicated. Based on the information, it would have been expected the total number of participants on ART would be 111. This is further confirmed by the data on class of ART in Table 3 which indicates 111 study participants. Could the authors explain this anomaly. | The authors are grateful to the reviewer for bringing this up. The correction has been done on Table 3 - Demographic Characteristics of Study Participants regarding ART and the figure **112** has been changed to **113** in the revised manuscript. It should be 113 and not 111 because the duration of **0 to 24** months for the ART will take care of the 2 patients who were naïve (zero duration). | Line189 and page 10 of the revised manuscript. |
